# Moringa as a household water purification method – community perception and pilot study in Guinea-Bissau

**DOI:** 10.1186/s12889-022-14344-w

**Published:** 2022-10-21

**Authors:** Aducabe Bancessi, Rosa Teodósio, Elizabeth Duarte, Aladje Baldé, Luís Catarino, Teresa Nazareth

**Affiliations:** 1 Nova School of Business and Economics, Nova University of Lisbon, Campus de Carcavelos, Rua da Holanda, n.1, 2775-405 Carcavelos, Portugal; 2 Unidade Clínica Tropical, Instituto de Higiene e Medicina Tropical, Universidade Nova de Lisboa, R. da Junqueira 100, 1349-008 Lisbon, Portugal; 3 Centro de Malária e Doenças Tropicais, Instituto de Higiene e Medicina Tropical, Universidade Nova de Lisboa, R. da Junqueira 100, 1349-008 Lisbon, Portugal; 4 Department of Sciences and Engineering of Biosystems, Institute of Agronomy, University of Lisbon, Tapada da Ajuda, 1349-017 Lisbon, Portugal; 5 Universidade Jean Piaget Guiné-Bissau, Campus de Antula, Bissau, Guinea-Bissau; 6 Centre for Ecology, Evolution and Environmental Changes (cE3c), & Global Change and Sustainability Institute (CHANGE), Faculty of Sciences, University of Lisbon, Campo Grande, 1749-016 Lisbon, Portugal; 7 Unidade de Parasitologia Médica, Instituto de Higiene e Medicina Tropical, Universidade Nova de Lisboa, R. da Junqueira 100, 1349-008 Lisbon, Portugal; 8 Faculdade de Medicina, Universidade Católica Portuguesa, Campus de Sintra, Estr. Octávio Pato, 2635-631 Rio de Mouro, Portugal

**Keywords:** Drinking water, Belief, Perceptions, Moringa, Water treatment

## Abstract

**Background:**

Public perceptions of water-related issues are still under-researched topics. The current paper intends to explore a local community’s perceptions regarding household water purification (HWP) strategies, namely before and after trying a new method: moringa seeds powder (moringa-teabag).

**Methods:**

In September 2020, six focus group discussions (*N* = 65) assessing perceptions about the usefulness of *Moringa oleifera* Lam (Moringaceae) as a HWP method (before moringa-based HWP trials), and questionnaires (*N* = 104) evaluating successes and identifying difficulties (after one week of moringa-based HWP trials). Participants were all women aged over 18 years, living in Ondame, Biombo region, Guinea-Bissau. Data were analyzed using qualitative and quantitative approaches.

**Results:**

The focus group discussions revealed that people are aware of the fact that water can transmit diseases. Although certain persons showed concern about shallow well water safety, people generally underestimate the risk, as they trust tubewell water. Not everyone had an understanding of what water contamination is, or the concept of medical importance. Some respondents declared they use traditional methods such as boiling and bleach to treat water before drinking. However, those who reported no kind of treatment indicated reasons such as lack of time, cost, and bleach’s taste and smell. In the questionnaire, more than half of the participants (68%) reported treating water before consumption. Nevertheless, these results are not consistent with our field notes. Participants demonstrated a strong belief in the capacity of moringa-teabags to purify water and even consider them better or much better (81%) than other methods. Participants asked for more information on moringa-teabag for household water purification.

**Conclusion:**

More information on water treatment and water safety would help to raise public awareness about waterborne diseases. These findings could be used to promote greater adherence to moringa-based HWP as an alternative to household water treatment.

**Supplementary Information:**

The online version contains supplementary material available at 10.1186/s12889-022-14344-w.

## Background

Safe and readily available water is a public health priority, whether it is used for drinking, domestic use, food production, or recreational purposes [1]. Historically, Sub-Saharan Africa experienced recurrent epidemics of waterborne diseases such as cholera and other diarrheal diseases [2–4]. In Guinea-Bissau, West Africa, outbreaks of cholera are common, although they have not been officially documented since 2013 [5, 6]. It is estimated that 82% of water for domestic use (drink, bath, and cooking) in the country presents fecal contamination, indicated namely by *Escherichia coli* [7]. Residents in rural areas seek alternatives to absent water systems, such as tubewells and shallow wells, to meet daily demands of quality water. A shallow well consists of a hole (≈ 200 cm in diameter) that has been dug, bored, driven, or drilled into the ground to extract water [8]. A tubewell (also called a borehole) consists of an iron pipe (15 cm in diameter), bored into an underground aquifer to extract water using a hand pump [9]. These two types of water sources are often contaminated due to the unsafe distances between groundwater-based water wells and pit latrines, frequently found in these rural areas. The engagement of local communities in prevention practices, namely household water purification (HWP), and water, sanitation, and hygiene (WASH) are crucial to avoid waterborne diseases. Examples of effective HWP techniques are bleach (i.e., addition of diluted chlorine solution to disinfect water), or water boiling. Although such methods are employed, their quality and reliability are uncertain, as HWP practitioners remain exposed to waterborne pathogens at home. This may be due to the low effectiveness of those methods, as well as to the consistency of their use (also mentioned in the literature as adherence or compliance). Other drawbacks of these practices have been documented such as the high costs of finding fuel and boiling and cooling water, the risk of burns from cooking fires among young children in developing countries [10], indoor air pollution and acute respiratory infections [11], the contribution of black soot from cooking fires to greenhouse gases [12], and lack of residual protection against recontamination of water during storage [13]. Moreover, chlorination is associated with chlorine taste and smell, and also with the inaccessibility of this treatment product [14].

*Moringa oleifera* Lam (Moringaceae), hereafter referred as moringa, has recently been recommended for water treatment [15], especially in developing countries, since other chemicals used in water purification are expensive [16]. Due to its numerous properties, this plant has several applications, for example in pharmaceutical, cosmetics, and food nutrition industries. For many years, several studies have been investigating the potential of its seeds and seed husks in drinking water treatment [17–19]. Coagulation/flocculation is the most investigated water purification process involving the use of moringa seeds. Evidence shows that it is a natural coagulant, highly efficient, cheap, and environmentally friendly. Recently, studies have been increasingly intensified mainly to understand how the coagulation mechanism occurs, to identify the proteins that are coagulating agents, and to develop isolation techniques [19, 20]. New methods, such as functionalization with nanoparticles [19], have been explored to enhance the removal of contaminants in coagulation/flocculation.

A considerable amount of research has focused on attempting to understand how water quality is perceived by the communities [21]. Findings suggest the need to effectively involve communities at important stages of implementation to ensure the long-term success of water quality interventions, due to their inability to recognize its benefits for health. This is further aggravated by unwillingness to pay for intervention maintenance, faulty perceptions on water treatment, lack of knowledge about health hazards associated with drinking unsafe water, false sense of protection from locally available water, resistance to change of taste or odor of water, and lack of support from male members of the household [22–24]. Evidence has been revealing other determinant factors for health-related behavioral change namely how individuals perceive their health risk (risk perception), the effectiveness of the proposed behavior (action outcomes), and their own facilities/ability to perform it (self-efficacy), among others [25–27]. The Essential-Perception analysis (EP-analysis) identified five essential topics for individuals adhering to a new health-related behavior (built for dengue prevention) that, when considered as a whole, revealed a correlation with the practices adopted [28, 29]. The present study adopted the rationale of EP-analysis to the prevention of waterborne diseases, aiming to assess essential/key perceptions for community engagement with moringa-based HWP. The current paper intends to explore those key perceptions of a local community in rural Guinea-Bissau regarding HWP strategies, namely before and after trying moringa-teabags as HWP. This will indicate whether the community is or not ready and responsive to these promising methods. Moreover the discrimination of which key perceptions are or not well consolidated within the community can support the selection of messages that need to be spread among the community if a moringa-based HWP is promoted in their area. The approach was based on the relationship between household water purification and household characteristics (such as age and literacy).

## Material and methods

### Study design

The research comprised two approaches: (i) a qualitative analysis using focus group discussions (FGD) before the moringa-based HWP trial (i.e., the participants had no previous experience with moringa-based HWP), to disclose perceptions on HWP strategies, including with moringa, and (ii) a quantitative analysis using a one-to-one questionnaire assessing perceptions regarding moringa as a HWP, following a one-week moringa-based HWP trial. FGDs provided the participants the opportunity to expand on any ideas, not offered in the quantitative questionnaire, that are critical to identify unsuspected obstacles to moringa-teabag use; in turn, the questionnaire allowed an objective assessment of the adherence to the proposed trial and the related perceptions [30].

### Studied Population

The study was conducted in Ondame (11°46′N, 15°56′W), a rural area in the Biombo region of Guinea-Bissau. Two reasons supported the selection of Ondame: (i) absence of a water supply system, (ii) geographical proximity and similarity to Quinhamel (rural area) – whose water quality data was previously reported, suggesting a high risk of fecal contamination in drinking water in Ondame [6], concerning epidemiologic data [5], type of water sources, ethnic diversity, and climate. Quinhamel was not chosen as the study area, because the drinking water quality was analyzed there and the topic was familiar to the residents, which could influence the results of the enquiries in the present study. To calculate the sample size, the Epitools platform was used – assuming an infinite population (since the authors could not count the population and no recent official data are available concerning individuals aged over 17 years) and that 90% of the villagers use moringa, a precision of the estimate of 5%, and a confidence level of 90%, a sample of 98 participants was obtained to which about 20% was added to fill a gap, in a total of 120 individuals in the sample. A minimum of 6 FGDs was assumed (one per village), and additional sessions would be planned if data saturation was not achieved. Only one FGD was allocated to each village in order to avoid contact between participants from different FGD; the physical distance between villages (more than 5 km) acts as a buffer. In the villages closest to each other (Dorse and Quinsana, Blom and Blim Blim, Quita-a and Sidja) all the FGD sessions took place on the same day. Three community health workers were involved in the process and established the first contacts with the community leaders of each village, as they are key stakeholders who directly oversee the functioning and health issues in all communities. Participants’ selection for the FGDs was proposed by the leader of each community by indicating around 20 women per village according to the study’s guidelines: women at least 18 years-old, in charge of the drinking water at home. Out of those 20 women, twelve were randomly selected to participate in each FGD, as recommended in the literature [31–33] and taking into account the possibility of withdrawals. For the questionnaire, the same sample was used, including both those who participated and those who did not participate in the FGDs, totalling 104 participants.

### Focus group discussion (before the moringa-based HWP trial)

#### Methodology

Six FGDs were conducted and audio recorded, to qualitatively assess communities’ perceptions. Participants were all at least 18 years-old. Each FGD lasted 30 to 45 min and written transcripts of the responses to the various questions were made (in addition to the audio-recordings). FGD sessions were conducted in a roundtable format gathering a minimum of 4 and a maximum of 12 individuals, as recommended [31–33]. Selected topics were discussed with a moderator until data saturation was reached or no new information was uncovered, in a local language (Guinea-Bissau creole). The moderator encouraged an open discussion, hoping that group dynamics would reveal deep feelings and thoughts. An FGD guide was used to direct the discussion (see supplementary material) containing a set of open-ended questions exploring various aspects such as the effects of unsafe water on health (e.i, are there diseases that are transmitted by water, or not?). The FGD guide was previously tested in a village that did not participate in the main study (Ondame) and modified according on the obtained feedback. The fieldworker was present as a facilitator during the meetings and interviews and took notes to document participants' comments relevant to the qualitative analysis. No information that could identify participants was retained in the transcription, the analysis or the subsequent report of findings.

FGD participation’s informed consent was given verbally and audio-recorded as part of the discussion since the level of literacy of the participants is very low. Participants in FGDs were asked confidentiality about all that was discussed in the sessions. The meetings were held at a time and place convenient to the participants, i.e., mostly in the mornings and in a village’s community hall. All participants volunteered to join the study. At the end of each session there was a socializing party, and this gathering of the participants was encouraged by the research team. The study was approved by the ethics committee (090/CNES/INASA/2020) from the National Institute of Public Health (INASA), Guinea-Bissau.

### Topics of interest for the qualitative approach

The Essential-Perception analysis (EP-Analysis) was validated in the dengue context, revealing a correlation between having the essential perceptions and adhere to the proposed preventive behavior (those selected assessed perceptions were thus considered essential for that behavioral adherence). In this study the Essential Perceptions Framework was adopted and adapted to the waterborne disease setting [28]. Following this methodology, five essential topics were maintained (Medical Importance, Local Risk perception, Household Water Purification, Water Contamination, and *moringa-based*—new HWP method) and 13 adapted essential perceptions (Table 1) were addressed based on the FGDs guide. The study was conducted from late September to the beginning of December 2020.

**Table 1:** EP-analysis essential topics and respective perceptions in the moringa-based setting and questions in FGDs

Themes	Perceptions	Questions
**Medical Importance**	Know that water can transmit diseases	*Are there diseases that we can get from water or not?*
	Present a correct example of a waterborne disease	*If yes, which one?*
	Mortality/ Morbidity	*Did you lose someone because of those diseases? What kind of damage can diseases got through the water do to us? (Which of those diseases has already happened to you?) Is diarrhea normal or a sign of illness? What damage can it cause to a person?*
**Local Risk Perception**	Safety of Ondame’s / own source of water	*Do you think Ondame's water is clean? In your opinion, is it contaminated or not? Talking specifically about the water you use, is it or is it not contaminated/clean?*
**Water Contamination**	Understanding contamination	*Do you know what water contamination is?*
		*What is the cause of the contamination?*
**Control Measures: Household Water Purification (HWP)**	Acknowledgment of HWP relevance	*Which way do you know to avoid getting diseases through water?*
	HWP practices	*At home, do you clean/purify the water? If yes, which ones?*
	Barriers/ Beliefs	*Who does not clean/purify the water? Why do you not do it? Do you not need it? Do you think water has no diseases? Do you think your water is not dirty? Is it expensive? You have no time for it? Is it too much work?*
**Control measures: Moringa-based—new HWP method**	Knowledge about moringa	*Do you know moringa (nené badadje)?*
	(Action-outcomes) / Belief in moringa as HWP	*If I tell you that it can treat water, what would you think? (Would it come as a surprise to you /or not)*
	Willingness to try	*If I gave you the moringa powder (nené badadje) to put in water for 5 min before consumption, would you accept to do it or not?*
	(Self-efficacy) Belief in own’s ability to perform	*Would you be able to continue doing it?*
	Barriers/ Beliefs	*What would be your main difficulty to implement water treatment with moringa?*

### Questionnaire (after the moringa-based HWP trial)

#### Methodology

A post-test-only design was performed, to assess perceptions one week after the moringa-based HWP trial. The fine moringa seed powder (moringa-teabag) was previously produced by Diocesan Caritas of Bissau. Then, moringa-teabags were prepared by us, each containing 2 g of moringa seed powder, to be diluted in 20 L of water (i.e., in a proportion of 100 mg/L) for 5 min, which has the potential to eliminate 99.9% of several pathogenic bacterial strains (*Escherichia coli, Pseudomonas aeruginosa, Salmonella typhi, Staphylococcus aureus,* and *methicillin-resistant Staphylococcus aureus (*MRSA) [34].

Each participant received seven moringa-teabags to use for one week, and the questionnaire was presented in the following week (late November 2020).

Questionnaires applied through one-to-one interviews were then used to extract in-depth information from 104 participants (65 from FGD plus those who did not have the opportunity to participate in the FGD) in Ondame’s villages. A semi-structured format was employed to collect data, exploring various aspects such as their belief in moringa's capacity to purify water (e.i., what was the general impression of using the moringa-teabags for water treatment?). Participation in the face–to–face questionnaire was based only on verbal consent since the level of literacy is very low. The surveys were performed by trained personnel (leading researcher) during October and beginning of November 2020. The study was approved by the ethics committee (090/CNES/INASA/2020) from the National Institute of Public Health (INASA), Guinea-Bissau.

### Topics of interest for the quantitative approach

The topics addressed concerned: General perception, Water sensory evaluation, Moringa adherence, and Comparison between moringa-based and other HWP (Table 2).

**Table 2:** The topics of interest in the moringa-based setting; and questions in questioner guide

Themes	Concepts	Questions
**Water treatment practices in the household**	Water treatment	*Do you treat the water?*
		*What is your main source of water?*
**General perception**	Satisfaction with moringa	*What is the overall impression on the use of the moringa teabags?*
		*How often have you used the teabags? Did you use the teabag in all the water consumed?*
		*If you did not always use it, why?*
**Water sensory evaluation**	Quality of the water	*When you put the teabag in the water, what did the water look like? Did the water change color? Did the water taste?*
**Moringa adherence**	Willingness	*Do you consider the use of moringa-teabag to treat water important? How easy/hard was its use? What was more difficult? If moringa becomes easily available would you use it in drinking water? Would you recommend it to others?*
**Comparison between moringa-based and other HWP**	Effectiveness	*Do you use another treatment method? If yes, which one?*
		*And how does it compare with the use of moringa?*

## Data analysis

### Qualitative data

Researchers coded the obtained data, as it is the most appropriate strategy to analyze FGD data [35], and then extracted it from the transcripts to conduct further analysis. The analysis was both deductive, when considering the previously defined topics (displayed in Tables 1,2), and inductive, when categories/topics emerged purely from the data. The research team critically reviewed each analysis product, ensuring analytic rigor and reliability by confirming that the data and illustrative excerpts were extracted to the correct domain. Discrepancies in data interpretation were discussed and resolved via consensus.

### Quantitative data

After the survey was completed, data was transferred to a secure server and a descriptive statistical analysis was carried out using SPSS v. 27.0 program SPSS Statistics software (IBM Corp., Armonk, NY, USA). A chi-square (χ2) and binomial tests [36–38] were applied to identify household characteristics leading to HWP acceptance and test dichotomous variables respectively. For all analyses, the level of significance was set at 5% (*p* ≤ 0.05).

## Results

The socio-demographic characteristics of the sample are described in Table 3. Of the 110 submitted questionnaires, a total of 104 fully completed ones were returned and analyzed. The remaining 6 questionnaires were not fully completed and were discarded from the analysis. Adding to the previously established ones, a new topic/category emerged from the data during FGDs: the main source of water (Table 3). Findings from both qualitative and quantitative analysis are presented below by topic.

**Table 3:** Socio-demographic characterization of the participants

	**Focus Group Discussion**	**Questionnaire**
	Inquired population	Inquired population
	(*N* = 65)	(*N* = 104)
	**n (%)**	**n (%)**
**Gender**		
Female	65 (100)	104 (100)
**Education level**		
Lower than high school	53 (94)	82 (90)
High school	12 (6)	22 (10)
**Occupation**		
Housewife	64 (99)	95 (91)
Student	1 (1)	9 (9)
**Age groups (years)**		
≤ 25	2 (3)	5 (5)
26–35	20 (31)	19 (17)
36–45	11 (17)	31 (31)
46–55	7 (11)	20 (19)
56–65	12 (19)	14 (14)
≥ 66	13 (20)	14 (14)
**Family household number**		
1–5	9 (14)	-
6–13	56 (86)	-

### Socio-demographic characteristics

Out of those 65 had previously participated in the FGDs sessions, comprising an average of ten participants (min. = 4, max. = 12). Demographic information concerning the respondent community is given in Table 3.

### Community perceptions before the moringa-based HWP trial

#### Medical importance

Participants showed awareness of the medical importance of waterborne diseases: they are conscious that water can transmit diseases and some were able to give a correct description of a waterborne disease. For example, *“some bacteria can be in the water and you do not know and you are going to drink that water and you get sick, but if you drink good water, you will not get diseases*” (FGD1, Int 3). Those who knew an example of waterborne diseases described “*diarrhea, vomiting and many diseases in the water*” (FGD3, Int 12). However, others were unable to name a correct example of waterborne disease “*well I do not know, it is you who will tell us* (FGD3, Int 9)”. Regarding mortality/morbidity, participants did not recognize the severity of waterborne diseases. They perceive the loss of family members due to illness, although they are not able to identify if that was due to water consumption, and in some cases they do not even know what the cause was – “*we may have lost family members and we do not know that it is because of the water, except when it was a cholera outbreak, then we knew that it was because of the water*” (FGD1, Int 13). On the other hand, they were aware that diarrhea is a sign of illness and that it can cause damage to our health – “*diarrhea is a very big disease*” (FGD1, Int 16).

### Local risk perception

The perception of water quality in Ondame varies according to the type of water source (tubewell or shallow well). Ondame’s tubewell water is perceived as cleaner and safer than shallow well water – *“the water from tubewell is better than from the shallow well, yes, because it is clean, while the water from the shallow well is dirty”* (FGD2, Int 20). One participant declared having no concern at all with the tubewell water – “*I have no concern with tubewell water and as we now have it within our reach and we are going to get it, and we spare the tiring boiling”* (FGD1, Int 21). Despite confidence in the safety of their water, namely from tubewells, some participants were aware of its potential contamination with rainwater – “*when it rains that cloudy, red water will go into the well*” (FGD4, Int 28). Yet, some raised concerns about the shallow water – “*when you are seeing an open shallow well water without lid, you know right away that it is not clean”* (FGD1, Int 23). Others consider that they drink shallow well water due to the lack of alternatives, even though they are aware that it can be dangerous (contaminated). Although some participants recognize a higher risk in shallow wells, some believe it is safe since their ancestors already drank that water. A participant shared her thoughts: *“you cannot say that this water we drink is not good, because since we were born, this is the water that we have been drinking, as we have not found any other pump (tubewell) here and this is the one we live with*” (FGD4, Int 18).

### Water contamination

Not all participants revealed an understanding of what water contamination is – “*I do not know*” (FGD6, Int 87), and some defined it as “*contaminated water, it is dirty water that is contaminated water*” (FGD5, Int 84). Nevertheless, they perceive contamination more as a transmission mode from person to person – “*contamination is when I have diarrhea and vomiting and if a get visits from my colleague, he/she can get sick through my illness*” (FGD3, Int 86). Participants correctly identify rainwater as the main cause of contamination – “*when it rains that cloudy, red water will go into the well*” (FGD4, Int 96).

### Household water purification (HWP)

In FGDs, a wide variety of comments arose regarding HWP. Respondents commented that they already use some traditional methods to treat water before drinking it – “*by bleach, or boiling or even lemon drop, or another if you have*” (FGD3, Int 47). Participants perceive that treating water at the home level is important and reported that they do it – *“I use a cloth to filter, when I cannot filter, I normally put a drop of bleach*” (FGD6, Int 41). For those who do not treat the water, the reason is that they feel safe – “*before we had the tubewell, we used to filter the water, but after there was the tubewell, I just wash the basin/bowl, clay pot, and lid very clean, and remove the water and put it into a clay pot and cover it with cloth*” (FGD3, Int 36).

Although participants mentioned that they treat water, they pointed out the barriers such as price, smell, time, and taste – “*As we do not have time for that, while we are working in the fields, we drink from shallow*” (FGD4, Int 52); another participant reported that “*some do not like to use bleach because of the smell, that is why they do not use it*” (FGD6, Int 55). In addition, one participant said: “*In fact it is very difficult for anyone here to put bleach in the water, it is just because of this quarantine (covid-19) that has arrived that people are now putting bleach in, if it was not for this it is difficult to see anyone putting bleach in the water*” (FGD6, Int 56). Participants perceive different needs to treat water according to their water source and aim. For drinking, they collect from tubewell, whilst a shallow well is used for cooking or bathing. The overall impression the participants gave was the need for a tubewell – “*if there is a tubewell, we avoid all that, it is just going and getting the water as it is not necessary to filter, but if you are tired and arrive at night and you are thirsty, you will get the water and drink it without filtering*” (FGD4, Int 58).

### Moringa based – new house water purification (HWP) method

Participants demonstrated knowledge about moringa, but in terms of food and medicinal aspects. They appeared to be surprised about its use as an alternative for traditional HWP, as they had never heard of it – “*yes, it is a surprise, as we have never done it and we do not even know about it and we have it here in abundance*” (FGD1, Int 73). They showed great readiness to accept the moringa-teabag as an alternative for HWP water purification at the household level – “*yes, we will because you have shown us that it can remove the bacteria*” *(*FGD2, Int 81). Several participants were very positive about their ability to carry out the process of using the moringa-teabags. They were interested in sharing information regarding the process, claiming that the main difficulty might be to implement the process. The request for information was consistent in all the groups – “*just tell us, if you explain to us and if it is something to help us, we will do it because what we want is to get rid of these diseases*” (FGD1, Int 80). There was also great interest in having a moringa-teabag to test the method – “*I think everyone will accept it because if there was no interest, we would not be here*” (FGD2, Int 76). Further, one participant illustrated that its future use would depend on the results from the first uses – “*if you bring it to us once and we feel it is good, even if you do not bring it anymore for us, we will keep doing it because we have it here*” (FGD4, Int 79). The examples of relevant citations for each topic of interest can be seen in the supplementary material (Tables S1 and S2).

### Community perceptions after the moringa-based HWP trial

#### Water treatment practices in the household

**Fig. 1:**
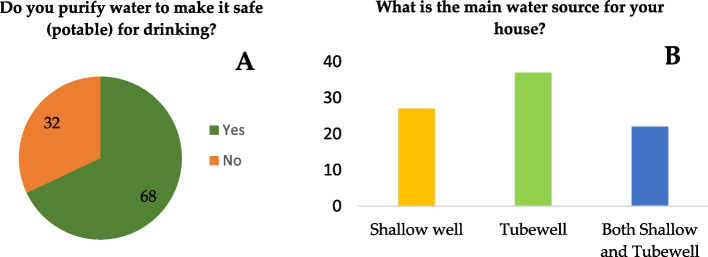
Percentage distribution of responses to questions concerning the purification of drinking water (**A**) and the main source of drinking water (**B**)

Over half of the households (68%) treat their drinking water before consumption; more than a quarter (32%) do not. The majority of the participants considered the tubewell as the main water source for drinking and other household uses (Fig. 1).

### General perception

**Fig. 2:**
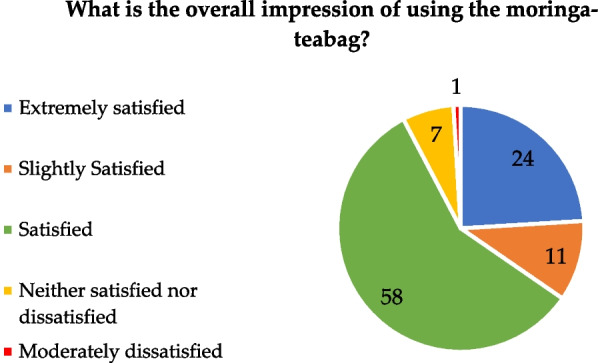
Percentage (%) of respondents' answers concerning overall impression about moringa-teabags

Belief in moringa can be defined by three broad categories: nutritional, medicinal, and chemical aesthetic attributes. In this study, the focus was on the chemical aesthetic attributes, and the participants were asked about their general impressions after using the moringa-teabag. Overall, households (93%) indicated that they were “Satisfied”, “Slightly satisfied” or “Extremely satisfied” with this water treatment(Fig. 2) However, 8% of households indicated that they were “neither satisfied nor dissatisfied" or "moderately dissatisfied”.

**Fig. 3:**
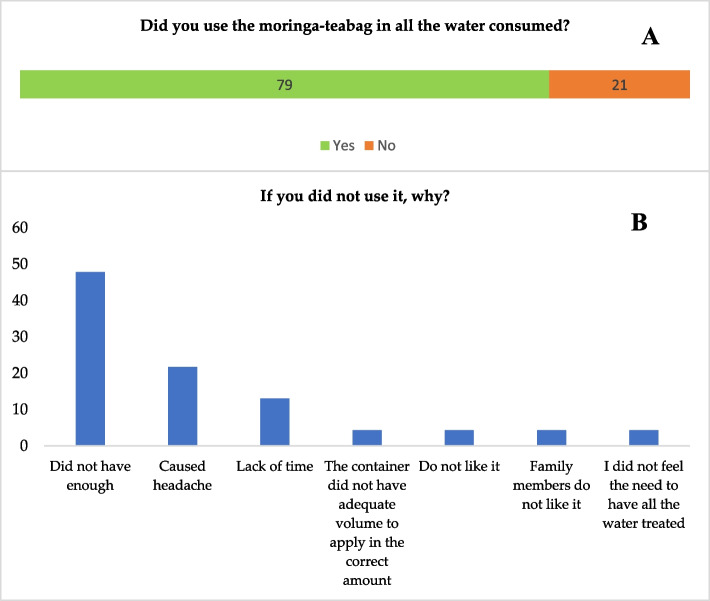
Percentage distribution of responses to questions concerning the use of moringa-teabag in all the water consumed **(A)** and the reason for not using it (B)

Figure 3 shows that 79% of the households used the teabag in all the water consumed. Among those who did not use it, 48% said they did not have enough, while 22% reported headaches after the first use, and 13% reported lack of time.

### Water sensory evaluation

**Fig. 4:**
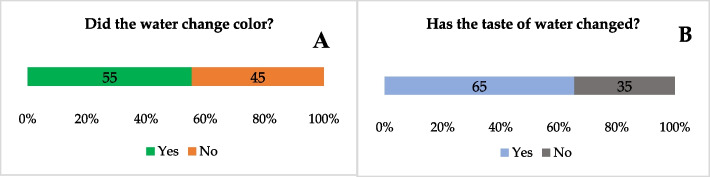
Percentage distribution of responses to questions concerning water sensory characteristics: **A:** change in color, **B**: change in taste

Respondents were asked to evaluate the quality of the moringa-teabag treated water, based on two sensory characteristics (color and taste). More than half of the households (55%) indicated that their drinking water color was changed; among these, 93% said the water became cloudy. Around 65% of the participants reported that the water taste was changed; it tasted either sweet (89%), sour (8%) or salty (3%) (Fig. 4).

### Adherence to moringa-based HWP

**Fig. 5:**
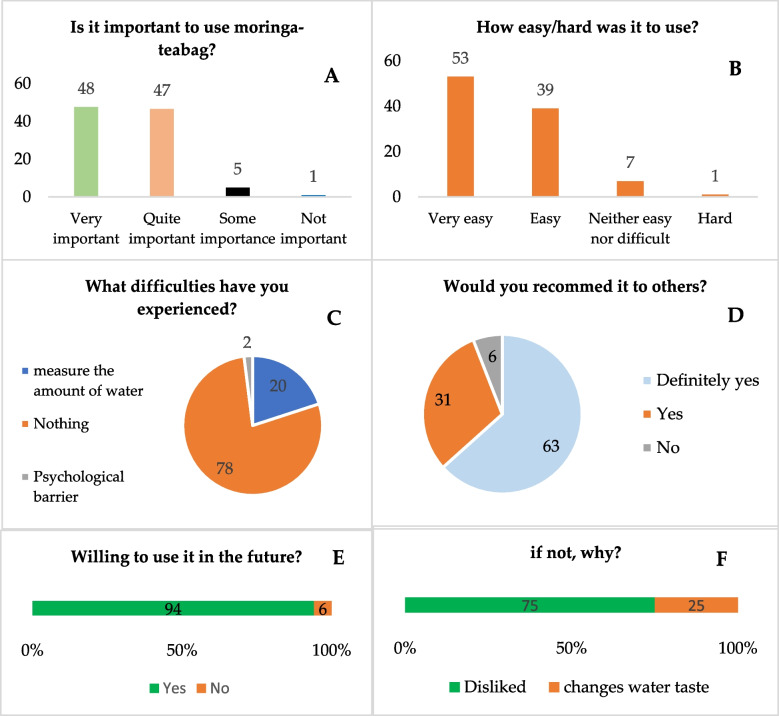
Percentage distribution of responses to questions concerning general appreciation (**A**), ease/difficulty to use the moringa-teabag (**B**), experienced difficulties (**C**), recommendation to others (**D**), willingness to use in the future to purify drinking water (**E**) and, if not, why (**F**)

Respondents were asked to rate the importance of using moringa-teabags for HWP, how easy/difficult was it, and what type of difficulties were found. The majority (95%) strongly believed that using moringa-teabag for HWP is very important or quite important, while the remainder said it is somewhat important or not important (Fig. 5). Most participants (92%) assertively mentioned that it was very easy or easy to use, in contrast with 7% that were uncertain, saying that it is neither easy nor difficult, and 1% for whom it was hard. The results show that the main difficulty found was related with measuring the amount of water for each teabag (20%), and psychological barriers (2%). When asked about the possibility of using the moringa-teabag in the future to treat water at home, 94% of the participants answered "yes" and 6% "no". For the latter, the main reason was that they did not like it (75%) or because it changed the taste of the water (25%). Regarding recommendation of the teabag as HWP, more than half (94%) said "definitely yes" or "yes"; 6% said "no" (Fig. 5).

### Comparison between moringa-based and other HWP

**Fig. 6:**
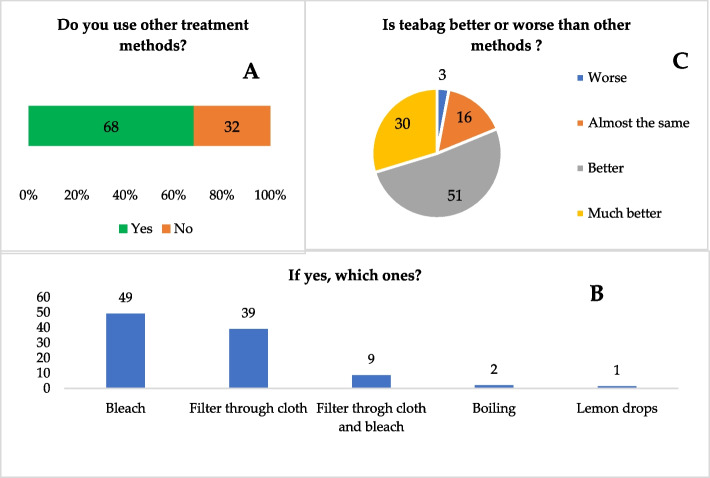
Percentage distribution of responses to questions concerning the comparison of moringa-based with other HWP: use of other methods (**A**), if yes, which ones (**B**), and which one is best (**C**)

Data on HWP practices were derived from three main core questions in the household survey (do you use other treatment methods, how does it compare with the use of the moringa-teabag, and would you recommend moringa-teabag to others). The findings show that (Fig. 6), among those who reported treating water for drinking, bleach was the predominant HWP method, used by over one-third of the surveyed households (49%), followed by filtering through cloth (39%) (Fig. 6). Other practices such as boiling or lemon juice drops were less common. Comparing moringa-teabag with other traditional HWP methods, the majority (81%) said that the former is better or much better than other methods, and 16% stated it is similar. Few households reported that moringa-teabag is worse (3%).

### Identifying household characteristics leading to HWP acceptance

The relationship between household water purification and household characteristics was analyzed using chi-square tests (χ2), while a binomial test was carried out to test dichotomous variables.

- *Research Hypothesis 1—The proportion of people who purify water before drinking equals that of people who do not (*H_0_); H_1_—The proportion of people who purify water before drinking is different from the proportion of people who do not. According to the results, the null hypothesis cannot be rejected (P > 5%) and it is concluded that the proportion of those who purify water before drinking equals that of those who do not (Table 4).
- *Research Hypothesis 2—The proportion of people who use water as soon as it is collected equals that of those who do not* (H_0_); H_1_—the proportion of people who use water as soon as they collect it differs from that of people who do not do that. The results showed that the null hypothesis is rejected, so we can conclude that there is no difference between the two groups (Table 4).
- *Research hypothesis 3 (relation of water treatment with schooling*).
  There is no difference between high schoolers and non-high schoolers regarding water purification practices (H_0_); H_1_—there is a difference between high schoolers and non-high schoolers regarding water purification practices. There was no difference between the two subgroups (*P* = 0.70) and, therefore, the literacy level does not influence the procedures concerning the purification of water before its consumption (Table 5).
- *Research hypothesis 4 (relation of water treatment practices with age)*.

**Table 4:** Binomial test for two independents variables

		**Category**	**N**	**Observed Prop**	**Test Prop**	**Exact Sig. (2-tailed)**
**Do you purify water to make it safe (potable) for drinking?**	Group 1	Yes	39	0.45	0.50	0.451
	Group 2	No	47	0.55		
	Total		86	1.00		
**Do you consume water as soon as you collect it from the well?**	Group 1	Yes	64	0.74	0.50	0.000
	Group 2	No	22	0.26		
	Total		86	1.00

**Table 5:** Crosstabulation of education level and age * water purification practices

			**Purify water for drinking**		**Total**	**X**^**2**^	***p***
		N (%)	Yes	No			
**Education level**	Lower than high school	69	32	37	69 100	0.149	0.700
	High school	17	7	10	17 100		
	Total	86	39	47	86		
**Age**	Young adults	39	16	23	39 100	0.538	0.463
	Adults	47	23	24	47 100		
	Total	86	39	47	86 100		

There is no difference between young adults and adults regarding water purification (H_0_); H_1_—there is a difference between young adults and adults regarding water purification. There was no difference between the two subgroups (*P* = 0.463) and, so, age did not influence the practices concerning the purification of water before its consumption.

A first analysis indicated some influence of age (years), with young people tending to purify water before consumption, but with a very tight statistical significance (*P* ≤ *0.05)*. In a second analysis, by considering two broad age groups (young adults and adults), the null hypothesis can be rejected with much more confidence (Table 5).

## Discussion

Qualitative and quantitative methods employed in this study allowed to gain insight into people's perceptions regarding the health effects of drinking water and household water purification practices in rural Guinea-Bissau households. Both approaches helped to identify key factors that influence the acceptance of the proposed methods, the water quality and its sustainability. Most traditional HWP methods (such as bleach or boiling) in the study area are reactive (in response to governmental announcements) rather than proactive (autonomous and well-planned by communities).

Several studies have confirmed that water conservation attitudes (WASH) and behaviors are closely related [39, 40]. The presented study indicates that the participants' belief in the medical importance of water is often biased. The nature of this bias seems associated to a ‘‘risk perception bias’’ and was noticed in participants who had some form of broad knowledge that contaminated water could cause health problems, and thus be more likely to find and report waterborne diseases. This was notorious in the case of a participant who presented a certain knowledge, given that her partner is a nurse.

For most of the participants, the concept of medical importance might not have been well understood. However, they know that water is a route for disease transmission, although few can name such diseases. They recognize the loss of a family member but do not know if it related to a waterborne disease, or to which one. As in the study carried out by Nazareth et al. [29] on the impact of a dengue outbreak on the preventive perceptions of the community from a temperate region (Madeira Island, Portugal), the participants did not show any perception of the medical importance.

The risk of well water differs, depending on the type of source (tubewell or shallow well). In general, respondents perceive that Ondame's tubewell water is safe, and show a high appreciation for tubewell water quality. Similar perceptions have been reported by Wu et. al [41] in their study on the impact of tubewell access and tubewell depth on childhood diarrhea in Matlab, Bangladesh. The natural filtration and the tubewell structure associated with groundwater supplies could be the main reason for the participant’s confidence in the safety of tubewell water. Drinking water stakeholders (companies, governments, and WHO/UNICEF) in Ondame (Guinea-Bissau) should inform the community about water quality issues such as safety and risks. Effective risk communication is complex and requires a thorough understanding of the information needs and risk perception of the general public [22, 42].

Household water purification is an important and effective part of the multi-barrier approach to ensure water quality and safety [43, 44]. Participants in FGDs declared they treat the water for drinking. However, this is not consistent with our field notes and even with some observations of the participants in FGDs who reported that they only treat water when they are recommended by community health workers. But, on the other hand, these results were in line with those from the questionnaire, where 68% of the participants reported using other methods to purify water at the home level. This difference in results could be explained by the possibility that participants consider cloth filtration as an effective treatment method. It is important to mention that from our observations and field notes, and even though they consider tubewell water safe, they normally use cloth to filter the water before putting it into the clay pot. Nevertheless, Roche et. al [45], studying public perceptions and improved water safety, reported that the respondents treated their drinking water using an in-home treatment method such as a filter. In FGDs, many participants indicated barriers to HWP practices. Sensory characteristics such as the smell and taste of bleach, and lack of time and money were the main reasons for not treating water at home.

In the questionnaire, the change in color or taste was also evidenced, although most participants considered that water’s taste improved after the use of moringa-teabag. A variety of treatment methods was reported, with almost half of respondents treating water with bleach followed by cloth filtering, or using more than one method. This result (cloth filtering) is also found in other studies on households depending on water wells (tubewell and shallow wells) [46–48]. However, the effectiveness of these treatments to reduce or eliminate microbiological hazards in water is questionable. The hypotheses tests showed that neither age nor literacy level influenced the water treatment practices in Ondame. This could be related to cultural issues (bias), such as the fact that they believe their grandparents drank this water and nothing bad happened to them.

Concerning moringa-teabag application, most participants believe in its capacity to purify water and show great willingness to use it for this purpose, considering it even much better than other methods and definitely recommending others to use it. This belief is supported by their knowledge of moringa as a medicinal and nutritionally valuable plant [49]. It should be noted that although there is a great belief in the moringa-teabag's effectiveness, some participants (30%) advised against it, claiming to have suffered from headaches after the first use, or that they or family members did not like it, or that they did not feel the need to have all their water treated, probably making it difficult to accept by these participants. Complaints about headaches may be linked to the high number and variety of bioactive compounds present in moringa seeds, although there is no evidence in the literature to support this side effect. Few studies on humans have analyzed the health benefits of moringa, but it seems to be well tolerated and to produce no side effects [50, 51].

The teabag for water purification can be an affordable and important practice to reduce waterborne diseases and to improve the quality of life for a large proportion of poor populations [52, 53]. The authors suggest the communication of water contamination levels to the communities [6], to raise their awareness of the actual water quality in their regions. Accurate risk perception can promote adherence to proposed methods of household water purification, such as the moringa-teabag. Another key step could be to involve influential actors, such as religious groups, local entities, and community leaders, in promoting commitment to these preventive practices.

However, it should be noted that during this study there were no outbreaks (history factor) that could jeopardize the study. The fact that respondents were only women (selection factor) does not influence the results, because women are the ones who take care of water at home. The study period (maturation factor) was not long, but the fact that two methodologies were applied and that the FGD participants also participated in the questionnaires may have influenced their acceptance of the use of the moringa-teabag. There was no loss of participants (mortality factor) during this study, but this is not a controllable situation and, over time, mortality might occur.

To sum up, this study provides three innovative pieces of information: *i)* community perceptions regarding waterborne diseases in Guinea-Bissau, *ii)* community perceptions regarding moringa-based HWP as a water treatment strategy, and *iii)* a successful pilot study on moringa use as a household water purification method.

## Conclusion

To date, there is no evidence in the literature reporting: *i)* community perceptions regarding waterborne diseases in Guinea-Bissau, *ii)* community perceptions on the use of moringa as a water treatment strategy, and *iii)* studies on actual moringa use as a household water purification method. This makes our findings pioneer/innovative and crucial due to the absence of sustained treated water supply or strategy in countries such as Guinea-Bissau, and to the disadvantages of the more common HWP methods. This study indicates that the participants were willing to try the moringa-teabag as HWP and after having tried it they were very satisfied with the results. Nevertheless, it is apparent that certain beliefs may influence some perceptions, such as the fact that they drink the same water for many years and think it is not contaminated, or the fact that they do not use chlorine because of the smell.

Correct perception of water contamination and risk are the key factors influencing water-use behavior. The majority of the respondents have misconceptions about water treatment, representing a high risk of waterborne diseases.

This paper demonstrates the importance of this new contribution to knowledge, providing detailed information to understand the correct perception of water contamination and risk correlated with water-use behavior. The results of this research can support the implementation of collaborative social innovation in order to understand how moringa seed powder will impact the environment and the social behavior along the water supply chain in rural areas. They can also be seen as new approaches to the practical objectives of identifying main barriers hampering the effective implementation of social innovation, and better understanding how moringa-teabags packaging innovation might impact the environment and various stakeholders across the water quality supply chain, especially as it relates to public health, infrastructure, cleaning of formal and informal water collectors, consumers, and policy.

These findings could be used to inform future campaigns to promote moringa as an alternative for household water treatment, in communities without water supply systems, with the potential for greater community engagement than current HWPs.

## Supplementary Information

Additional file 1: **Table S1** Sample of results from thematic analysis

Additional file 2:

## Data Availability

Analytic data are available in supplementary file

## Abbreviations

HWP: Household water purification, water
WASH: Water, sanitation, and hygiene
FGDs: Focus groups discussion
INASA: The National Institute of Public Health
SPSS: Statistical Package for the Social Sciences
