# Supplementary material for: Moringa as a household water purification method – community perception and pilot study in Guinea-Bissau
Source: BMC Public Health. 2022 Oct 21;22:1953. doi: 10.1186/s12889-022-14344-w (PMC9587536; doi:10.1186/s12889-022-14344-w)
Supplement: Supplementary file 1 — Additional file 1: Table S1 Sample of results from thematic analysis [file 12889_2022_14344_MOESM1_ESM.docx]

| Themes | Concepts | Quotations |
| --- | --- | --- |
| Medical Importance | Know that water can transmit diseases | *"In the shallow well, there are bacteria, and if you take the water out of the well and did not filter, you can then drink these bacteria and can get many diseases".* |
|  | Presented a correct example of a waterborne disease | *“We can get diarrhoea and stomach pain”.* |
|  | Mortality/morbidity | *“You could die, even though we do not know if it is from the water”.* |
| Local Risk perception | Safety of Ondame’s/ own source water | *“When you see a well (shallow well water) open without a lid, you know right away that it is not clean”.*  *“The water may be clean and contaminated”.* |
| Household water purification | Acknowledgement of HWP relevance | *“In our house we treat, we all boil the water before drinking it”.* |
|  | Barriers/beliefs | *“Due to tiredness, because if you leave tired from work, you draw the water and drink it, even if you draw it in the well, you will drink it”.* |
|  |  | *“In fact, it is very difficult for anyone here to use bleach, because of this quarantine that has arrived, people are now putting it, if it was not for that, it is hard to see someone put the bleach in the water”.* |
|  | The main source of water | *“If you have tubewell, you can even use it to cook, but if you are far from the tubewell, you will have to use the water from the shallow well”.* |
| Water contamination | Understanding Contamination | *“Contaminated water, it is dirty water that is contaminated water”.* |
|  |  | *“We understand it is because of the dirtiness”.* |
| *Moringa oleifera* – a new house water purification (HWP) method | Knowledge about moringa | *“We know it well”.* |
|  | Barriers/beliefs | *It will depend, if we keep all the information, we can do it, but if we do not it will be difficult* |
|  | Action-outcomes | *“Yes, because we have already seen what it does”.* |
|  | Availability to try | *“Something you have never seen is that may you refuse to do, but after what we have seen here, we will not refuse to do it”.* |
|  | Self-efficacy | *“Yes, we will be”* |
|  | Support future plans | *“Yes, we will, because you have already showed us that it can remove the bacteria”.* |

Table S1 – Sample of results from thematic analysis
